# Neurovascular Grafting in Complex Upper Extremity Trauma: A Case Series Report

**DOI:** 10.7759/cureus.69467

**Published:** 2024-09-15

**Authors:** Hugo E Beyuma-Mora, Juan C Morales-Gonzalez, Eduardo Zavala-Elizondo, Veronica I Villalobos-Salazar, Jaime I Ruelas-Pérez

**Affiliations:** 1 Plastic and Reconstructive Surgery, Instituto Mexicano del Seguro Social, Unidad Médica de Alta Especialidad, Hospital de Traumatología y Ortopedia No. 21, Monterrey, MEX; 2 Trauma and Orthopaedics, Antiguo Hospital Civil de Guadalajara, Guadalajara, MEX

**Keywords:** graft, nerve, nerve injury, trauma, upper extremity, vascular injury, vein

## Abstract

Upper extremity trauma is one of the most frequent surgical emergencies encountered in hospital trauma units. The complexity of injuries to the hand and forearm is due to the convergence of multiple anatomical structures within a relatively small and compact area, all of which are essential for the proper function of the extremity. Among these, neurovascular injuries are particularly significant. While these lesions are rarely associated with high mortality, inadequate or delayed management often results in severe dysfunction. In this paper, we present a series of cases involving complex forearm and upper extremity trauma, where autologous neurovascular grafts were utilized under microsurgical techniques for reconstruction.

## Introduction

Up to 20-40% of polytraumatized patients presenting to the emergency department exhibit associated upper extremity injuries, with fractures and multiple soft tissue lacerations being the most common [1-3]. In our population, these injuries predominantly occur to workers and laborers within factories and the textile industry, primarily as a result of blunt and crushing trauma, as well as from assaults by third parties, including penetrating trauma, lacerations, and gunshot wounds.

Peripheral vascular injuries of the upper extremity, particularly involving the axillary and brachial arteries along with their branches, account for 40 to 75% of all vascular injuries treated in civilian trauma centers. The anatomical distribution of these vessels renders them particularly susceptible to both blunt and penetrating trauma. The primary complications of such injuries include lethal exsanguination, multiple organ failure, and the potential loss of the extremity distal to the injury [4].

Peripheral nerve injuries in the upper extremity are also highly prevalent, with the most common mechanism being laceration by sharp metal objects or machinery. These injuries often result in significant functional impairments in the hand and the rest of the extremity [5-6].

The initial approach to these injuries is guided by the ATLS (Advanced Trauma Life Support) protocol, which includes a rapid medical history with a focus on the time since injury, mechanism of injury, and associated comorbidities. This is followed by a thorough physical examination of the affected extremity, including an assessment of the condition of exposed tissues, identification of severed or lacerated structures, a distal vascular assessment, and evaluation of sensation and motor function [7-8].

If the patient's condition permits, a surgical assessment should be conducted under appropriate anesthesia, along with initial debridement under hemostatic control to allow for complete visualization of the anatomical structures involved [7-10]. Following the initial assessment, the critical decision of whether to salvage or amputate the limb must be made. Established scoring systems, such as the Mangled Extremity Severity Score (MESS), are instrumental in guiding this decision-making process, following the principle of "life before limb." The ischemia time for the upper extremity, which is generally established at approximately 6 hours, is a key determinant in the feasibility of limb preservation [4, 7, 8, 11-13].

Once the viability of the upper extremity has been confirmed, a detailed reconstruction plan should be formulated [14]. Various approaches and algorithms are available in the literature; however, the protocol proposed by Mahajan et al. most closely aligns with the procedures performed in our unit: 1) assessment of tissue perfusion, 2) application and inflation of sterile tourniquets, 3) radical debridement, 4) bone shortening and fixation, 5) muscle/tendon repair, 6) release of tourniquets, 7) definitive vascular repair, 8) nerve repair, and 9) temporary or definitive skin coverage [7].

During the vascular assessment of the extremity, the absence or reduction of distal pulses, a positive Allen test, and the presence of "hard signs" of vascular injury, such as active or pulsatile bleeding, pulsatile or expansive hematoma, clinical signs of ischemia, or a murmur or thrill suggestive of an arteriovenous fistula, as well as the precise identification of the anatomical site of vascular injury, are clear indications for immediate surgical exploration and reconstruction [4, 7, 8]. Vascular injuries classified as Type 2 or 3 (complete wall defects with pseudo-aneurysms or hemorrhage, and complete transection with bleeding or occlusion) also warrant surgical intervention. Vascular repair may be performed using direct (end-to-end) repair, venous patch plasty, or, in cases of more extensive lesions, interposition of autologous or prosthetic venous grafts [4, 14-16].

In cases of forearm injuries, both the radial and ulnar arteries should be repaired, despite the hand's ability to survive with only one of these arteries intact. Repairing the primary veins is also important, as it helps reduce postoperative edema and maintain the patency of the arterial repair. When primary repair is not feasible, venous grafts provide a suitable alternative [7, 17].

The initial peripheral neurological assessment includes both motor and sensory evaluations. According to the Seddon and Sunderland classifications, "axonotmesis" (Sunderland type 4) and "neurotmesis," which correspond to a complete disruption of the nerve, require surgical management. This management may involve direct end-to-end repair or the use of nerve grafts if the defect is large or if there is excessive tension on the repair. Alternative strategies include the use of autologous non-nerve grafts, biodegradable nerve conduits, support cells, growth factors, extracellular matrix, and nerve transfers [5, 18-20].

## Case presentation

Case 1

**Figure 1:**
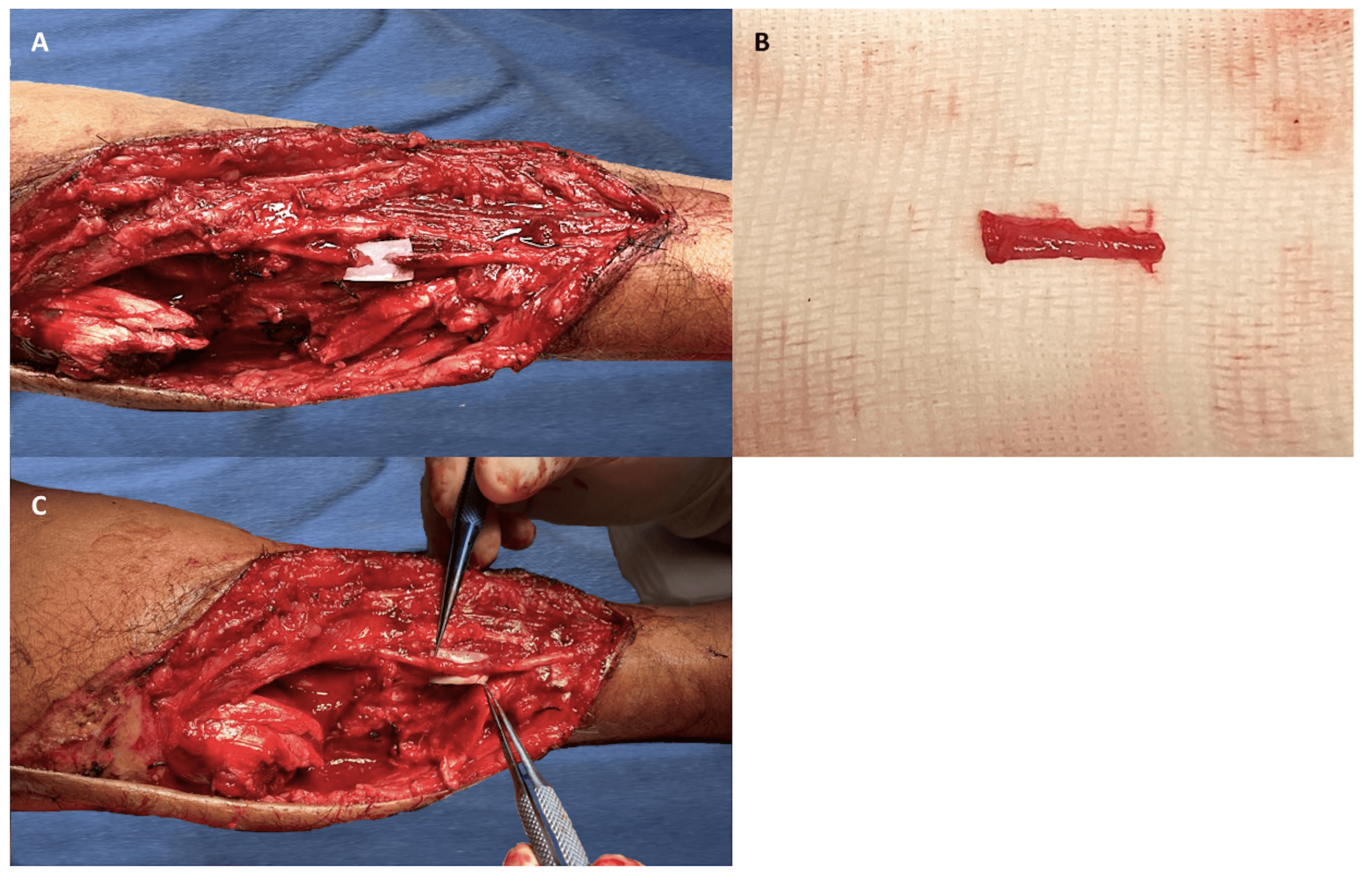
(A) Complete transection of the superficial branch of the radial nerve with a 1 cm defect. (B) Harvested cephalic vein graft. (C) Cephalic vein graft utilized as a conduit scaffold for nerve repair.

A 38-year-old male patient sustained blunt-cutting trauma from a machete strike to the radial border of the middle and distal thirds of the left forearm following an altercation. Despite the lack of distal vascular compromise and an intact motor examination, the patient exhibited anesthesia in the sensory branch territory of the radial nerve. Surgical exploration revealed myotendinous damage to the brachioradialis and a complete transection of the superficial branch of the radial nerve with a 1 cm gap, as illustrated in Figure 1A. The cephalic vein was dissected, and an autologous graft was harvested. This graft was then utilized as a conduit, placed using a microsurgical technique that involved a "sleeve" approach where the adventitia was sutured to the epineurium using interrupted 10-0 nylon sutures, as depicted in Figure 1B and Figure 1C. The procedure was completed with a tenorrhaphy of the brachioradialis.

Case 2

**Figure 2:**
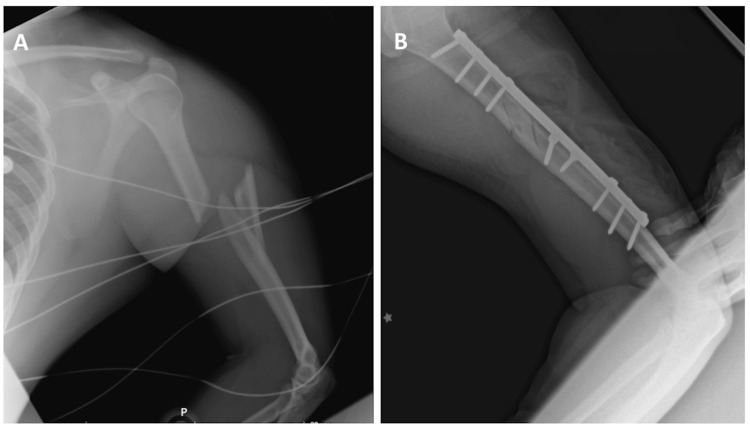
(A) Fracture of the left humerus at the middle third involving the radial groove. (B) Post-operative view following open reduction and internal fixation using osteosynthesis materials.

**Figure 3:**
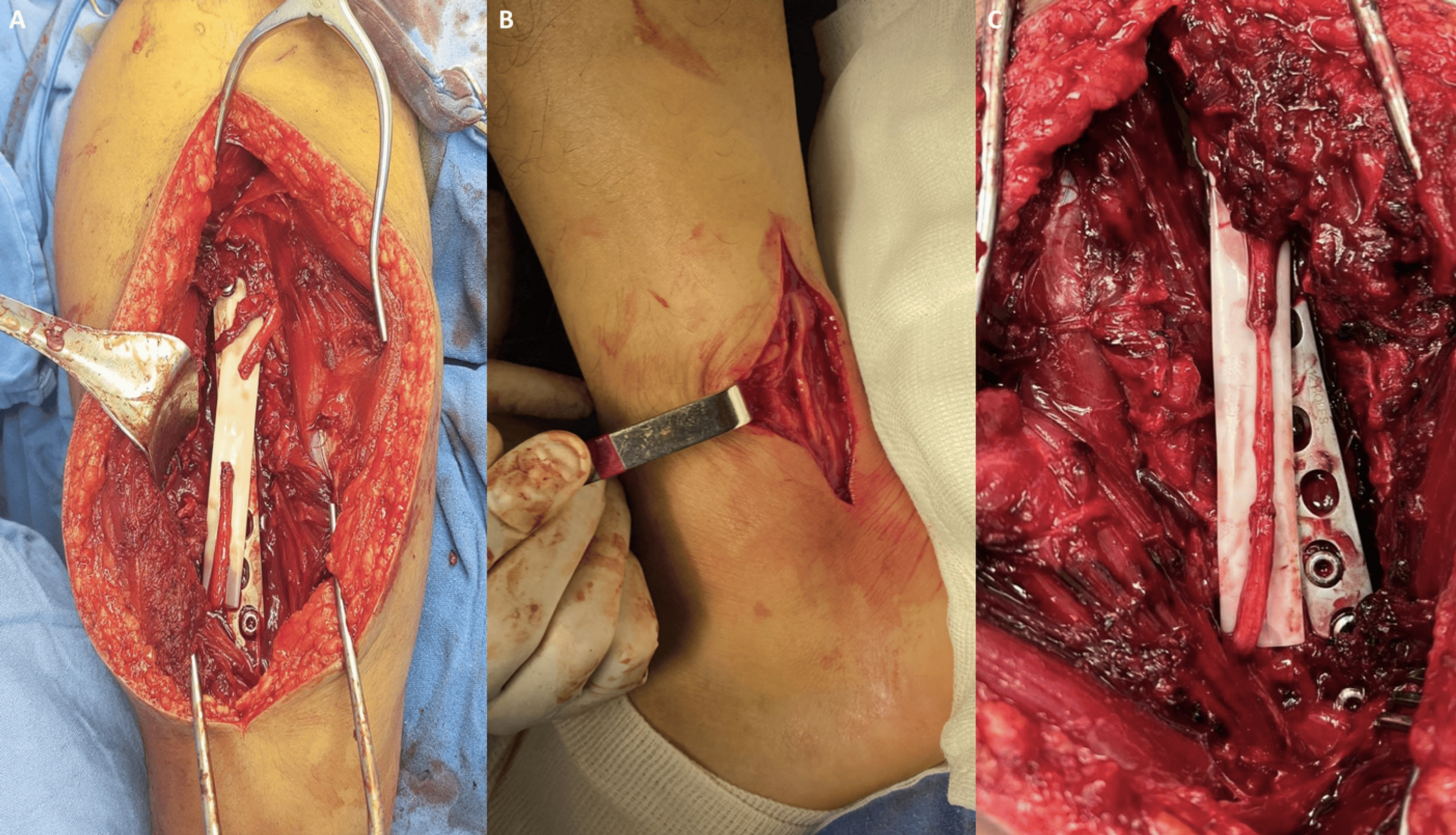
(A) Radial nerve with a 3 cm defect at the posterior aspect of the humerus. (B) Donor site for the sural nerve graft. (C) Sural nerve graft used to restore continuity of the radial nerve.

A polytrauma male patient in his early thirties sustained multiple fractures, including a fracture of the middle third of the left humerus, in a car accident, as shown in Figure 2A. During the open reduction and fixation with osteosynthesis materials (plates and screws), as depicted in Figure 2B, a high and complete transection of the radial nerve was identified at the level of the humeral radial groove, resulting in a 3 cm gap, as seen in Figure 3A. An autologous nerve graft was harvested from the sural nerve of the contralateral lower extremity, as shown in Figure 3B. This graft was applied using a microscopic microsurgical technique with interrupted 10-0 nylon sutures, as seen in Figure 3C. The reconstruction was finalized with subsequent myorrhaphies to protect the nerve repair from contact with the osteosynthesis plate, followed by layered wound closure.

Case 3

**Figure 4:**
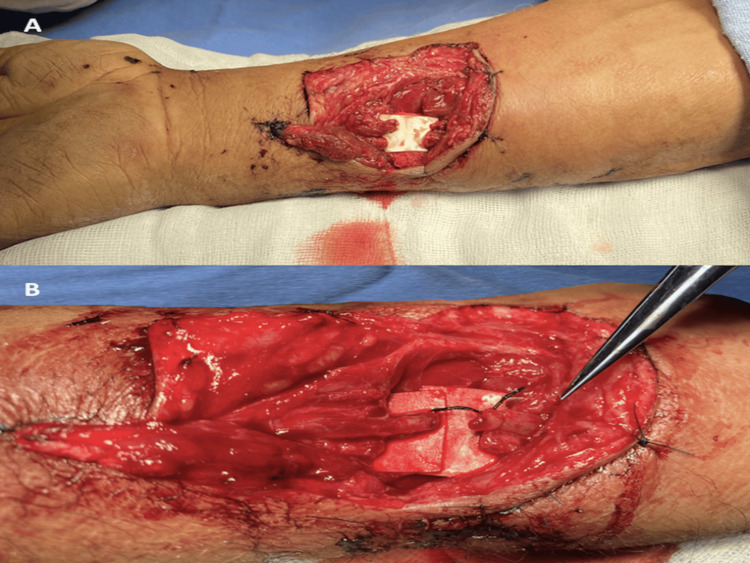
(A) Laceration of the ulnar neurovascular bundle. (B) Identified vascular and nerve defects, with the ulnar artery previously ligated.

**Figure 5:**
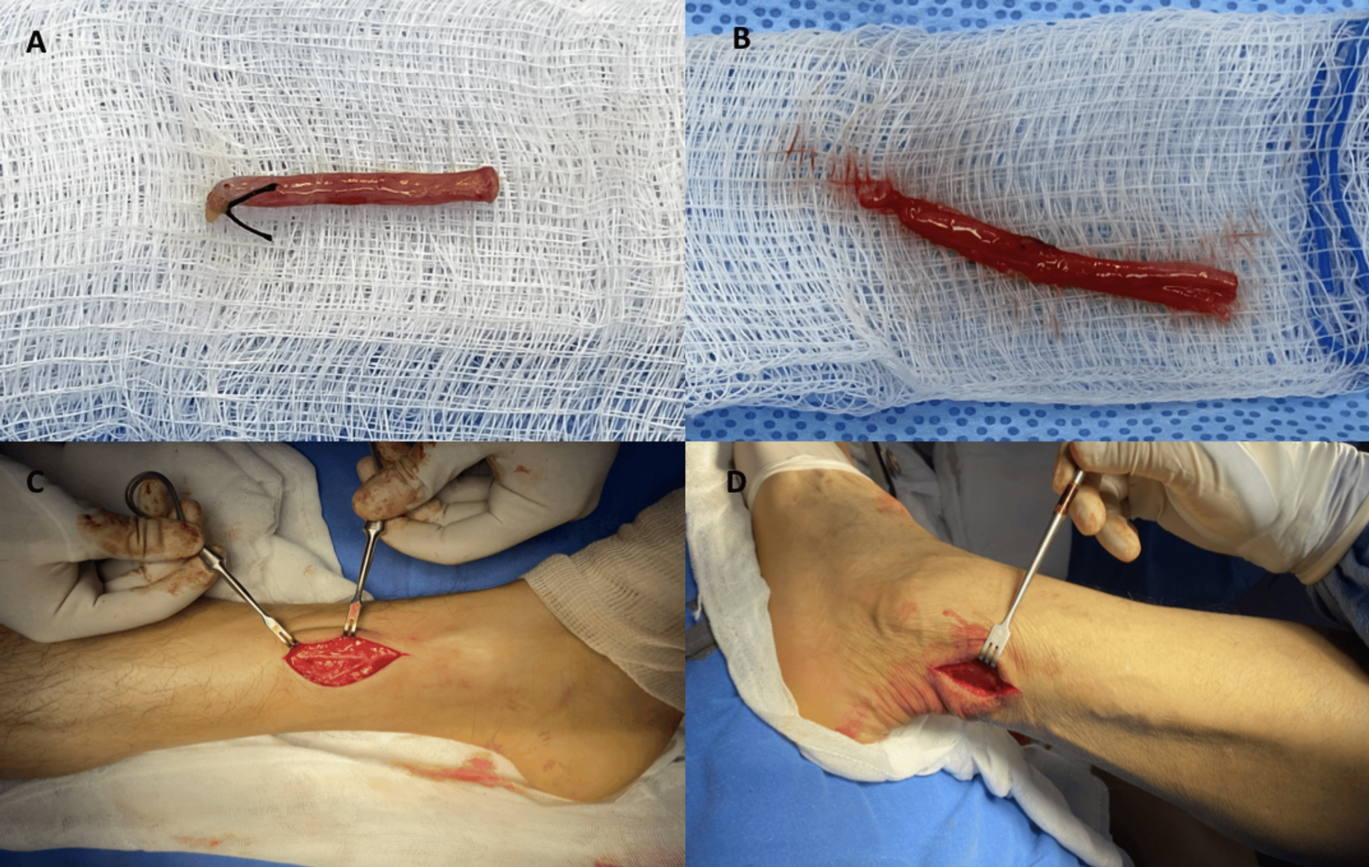
(A) Harvested great saphenous vein graft. (B) Harvested sural nerve graft. (C) Donor site of the great saphenous vein. (D) Donor site of the sural nerve.

**Figure 6:**
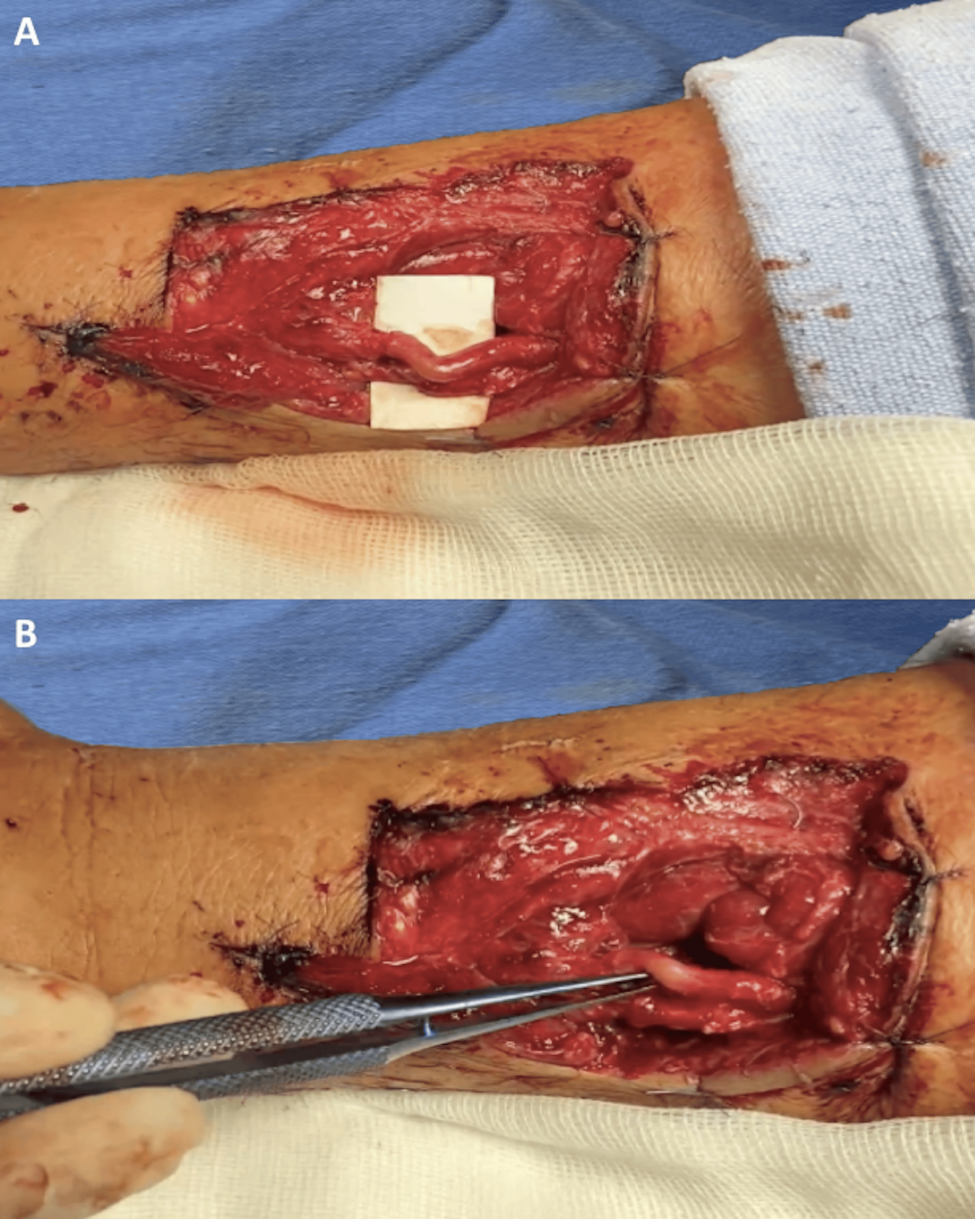
(A) Saphenous vein and sural nerve grafts reestablishing continuity of the ulnar neurovascular bundle. (B) Patent vein graft confirmed by the presence of an arterial pulse.

A 53-year-old male sustained a work-related injury involving cutting trauma with a steel blade to the ulnar border of the middle third of the right forearm, resulting in damage to the ulnar neurovascular bundle. At the initial care center, ligation of the ulnar artery was performed to control hemorrhage, and the patient was subsequently referred to our specialized unit. Upon surgical assessment and debridement, a myotendinous section of the flexor carpi ulnaris, along with a 1 cm vascular gap and a 2 cm nerve gap, were identified (as seen in Figure 4A-4B). A reversed venous graft from the great saphenous vein was harvested, as illustrated in Figure 5A and Figure 5C, followed by the harvest of a nerve graft from the sural nerve as seen in Figure 5B and Figure 5D. Both grafts were obtained from the contralateral lower limb. The neurovascular grafts were applied under microsurgical technique, with the vein graft being sutured using interrupted 8-0 nylon under vascular control with microclamps, and the nerve graft being secured with interrupted 10-0 nylon sutures, as seen in Figure 6A and Figure 6B. The procedure was completed with a tenorrhaphy of the flexor carpi ulnaris.

Case 4

**Figure 7:**
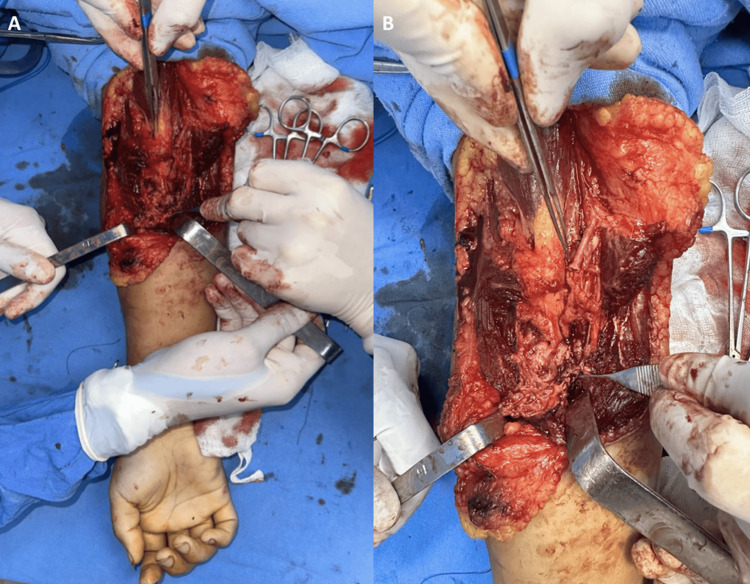
(A) Complex trauma of the upper extremity. (B) Involvement of the distal brachial artery, including its bifurcation and the origins of the radial and ulnar arteries.

**Figure 8:**
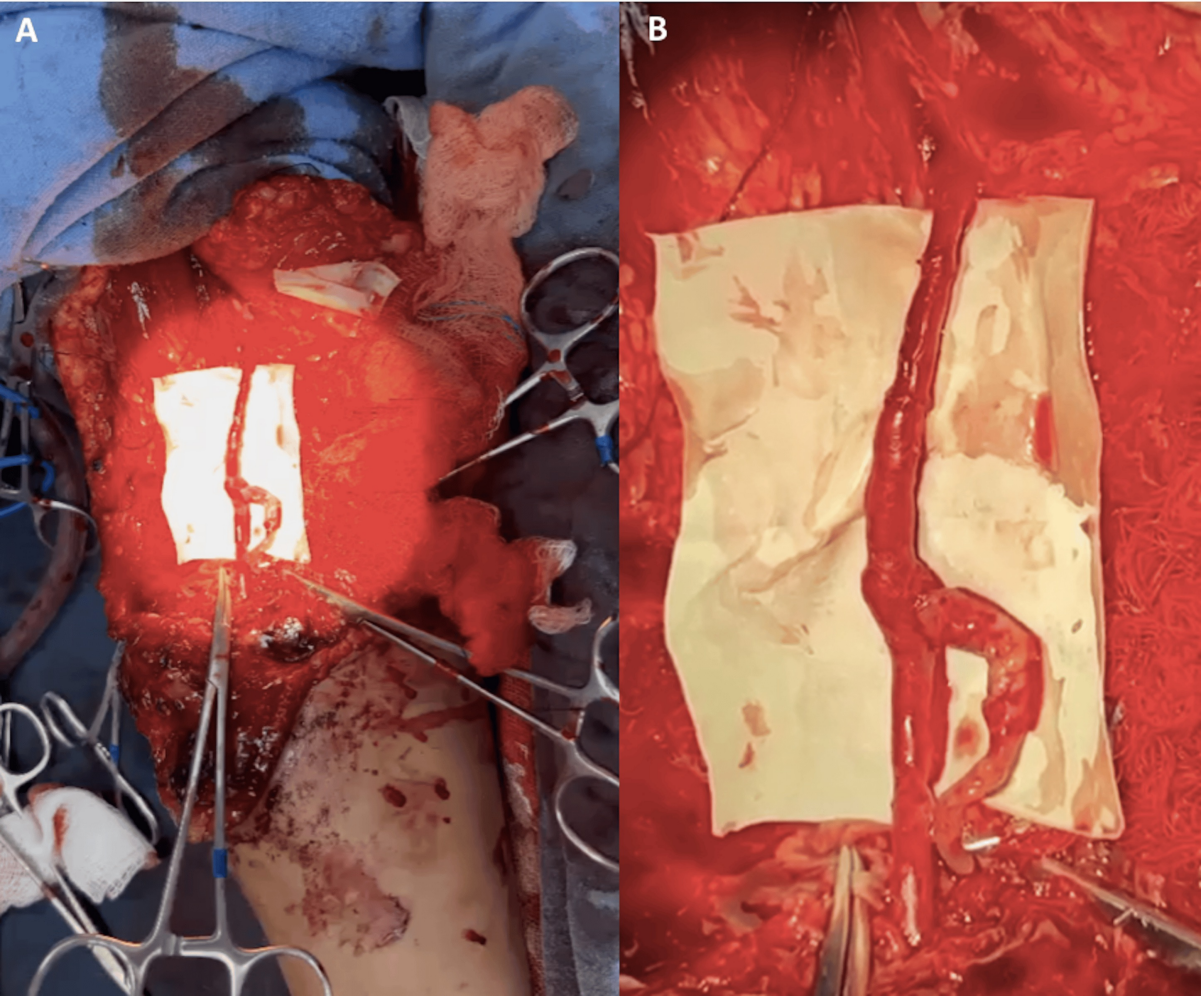
(A) Restoration of continuity of the brachial artery and its terminal branches. (B) Vascular graft from the basilic vein creating a new brachial artery bifurcation.

A male patient in his early thirties sustained a sharp trauma from glass during a workplace accident, resulting in a wound extending from the arm, across the antecubital fossa, to the proximal third of the right forearm, with distal vascular compromise, as depicted in Figure 7A and Figure 7B. Surgical exploration revealed a loss of vascular integrity from the distal third of the brachial artery, including its bifurcation and the origin of the radial and ulnar arteries. Reconstruction was performed under vascular control with a pneumatic tourniquet, utilizing vascular grafts harvested from the basilic vein of the ipsilateral upper extremity. An end-to-end graft was placed between the brachial and radial arteries using interrupted 8-0 nylon sutures, and a second end-to-side graft was placed between the brachial and ulnar arteries, also using interrupted 8-0 nylon sutures, to recreate the bifurcation under microscopic microsurgical technique. Vascular patency and the presence of a distal arterial pulse were confirmed, as seen in Figure 8A and Figure 8B.

## Discussion

The principles of microsurgical repair include adequate exposure of both the proximal and distal ends of the injured structures, dissection and isolation of the affected areas, and the use of magnification tools, such as surgical loupes or a microscope, to enhance precision. Non-absorbable, synthetic, monofilament sutures ranging from 8-0 to 11-0 are typically used in either continuous or interrupted patterns, depending on the specific anatomical defect. In vascular repair, the use of microclamps, local or systemic heparinization, and microscopic assessment of the vascular wall for intimal injury or thrombosis, followed by distal thrombectomy, are crucial steps. For nerve repair, meticulous debridement of visible ends, bone shortening, joint flexion, and intrafascicular dissection to gain length, with symmetrical alignment of the vasa nervorum and epineural or fascicular suturing are essential [5, 21].

Autologous venous grafts are preferred for the repair of arterial lesions in extremities due to their high patency rates and low incidence of secondary infection. When selecting a graft, factors such as availability, ease of dissection, and vascular diameter are considered. Common options include the greater and lesser saphenous veins, superficial femoral vein, and arm veins such as the cephalic and basilic veins. The saphenous vein is often the graft of choice due to its thicker vascular wall and availability, although arm veins are also suitable for arterialization despite their thinner walls [16, 22-25].

Nerve grafts serve as conduits that guide and scaffold axonal regeneration towards the distal nerve end, facilitating organ-terminal re-innervation. The gold standard for nerve repair, when direct repair is not feasible, is nerve autografting. Common donor sites for sensitive nerve segments include the sural nerve, medial and lateral antebrachial cutaneous nerves, dorsal cutaneous branches of the ulnar nerve, lateral femorocutaneous nerve, and superficial sensory branches of the radial nerve [17, 26-27]. There are also autologous biological alternatives to nerve grafting, including venous grafts and frozen striated muscle. These materials, being immunologically compatible and non-toxic, have been shown to promote axonal regeneration with minimal complications [28].

## Conclusions

The initial approach to complex upper extremity trauma requires a thorough physical examination to assess the extent and feasibility of the injury. The decision-making process regarding the appropriate reconstructive options depends on the damaged anatomical structures, the severity of the injury, the surgeon's microsurgical expertise, and the available resources. The morbidity associated with neurovascular injuries in the upper extremity, including the hand, can be significantly reduced with prompt and appropriate management, particularly when reconstruction is performed in the immediate post-injury period. Neurovascular grafts offer a viable reconstructive alternative when primary repair is not feasible.
